# Selective or sufficient? Revisiting forensic cardiac sampling in light of the seaport criteria

**DOI:** 10.1007/s12024-025-01128-y

**Published:** 2025-11-03

**Authors:** Graziano Domenico Luigi Crudele, Alberto Amadasi

**Affiliations:** 1 Department of Pathology, AULSS 9 Scaligera, Via Circonvallazione, 1, San Bonifacio, Verona, 37047 Italy; 2 Institute of Legal Medicine and Forensic Sciences, University Medical Centre Charité, University of Berlin, Turmstaße 21, Building N, 10559, Berlin, Germany

The recent article by Provenzano et al. [1] offers a significant contribution to the debate on the ethical and practical implications of organ preservation in forensic autopsies. However, its conclusions on the appropriateness of selective sampling, particularly of the heart, require further consideration in light of the new conse "http://e appropriateness of selective sampling, particularly of the heart, require further consideration in light of the new conse"nsus criteria developed by the Society for Cardiovascular Pathology (SCVP) and the Association for European Cardiovascular Pathology (AECVP): the so-called Seaport Criteria for Lymphocytic Myocarditis [2], which have broad forensic validity [3].

Provenzano et al. correctly recognize that myocarditis represents one of the most frequent histological diagnostic challenges in forensic cardiac evaluation. However, the view that even a representative cross-section might be sufficient in cases other than sudden unexpected death without evidence of coronary artery disease must be reinterpreted in light of current histopathological evidence. The Seaport Criteria [2] establish that, to reliably confirm or exclude lymphocytic myocarditis in autopsy specimens, at least six full-thickness ventricular sections must be examined. This requirement arises from the awareness that myocardial inflammation has a discontinuous distribution and that limited or unsystema "http://requirement arises from the awareness that myocardial inflammation has a discontinuous distribution and that limited or unsystema" tic sampling exposes a concrete risk of underdiagnosis. The subsequent complementary document by Fishbein and Preston [4]further clarifies that the purpose of the “Big Seaport” is to standardize the evaluation of large samples, ensuring uniformity and inter-institutional reproducibility.

The main point of tension between the indications provided by Provenzano’s work and the technical requirements represented by the Seaport criteria concerns the dichotomy between a minimum selective threshold (imparted by a principle of ethical selectivity according to Provenzano) and a minimum diagnostic threshold (imparted by a principle of representative rigor according to Maleszewski). For a methodologically reliable diagnosis or to exclude myocarditis, at least six full-thickness sections are required in an autopsy sample. The minimum selective threshold may not satisfy or be included in the minimum diagnostic threshold, especially since myocarditis is not always the cause of sudden death that would justify whole-heart preservation (see Sect. 3.2 of Maleszewski’s work).

We believe it is our duty at this time to foster the scientific debate on the newly developed Seaport criteria, which, especially in the forensic field, could play a fundamental role in the daily practice of heart sampling in relation to the assessment of myocarditis.

## Data Availability

The Authors confirm that the data supporting the findings of this study are available within the article.

## References

[1] Provenzano G, Pestourie L, Guilbeau-Frugier C, Telmon N, Saint-Martin P, Pefferkorn E. Impact of organ sampling for forensic pathology: should heart and brain be retained whole? Forensic Sci Med Pathol [Internet]. 2025 [cited 2025 Oct 18]; 10.1007/s12024-025-01074-9. 40932584

[2] Maleszewski JJ, Banner J, De Boer H, De Gaspari M, Fishbein MC, Parsons S, et al. Lymphocytic myocarditis: A histopathologic definition and classification from the Society for Cardiovascular Pathology and Association for European Cardiovascular Pathology. II: Surgical and autopsy specimens. Cardiovascular Pathology. 2025;78:107748. 10.1016/j.carpath.2025.107748. 40582474

[3] Sampson B, Maleszewski JJ, Michaud K. The seaport criteria for myocarditis. Forensic Sci Med Pathol [Internet]. 2025 [cited 2025 Oct 20]; 10.1007/s12024-025-01103-7. 41091450

[4] Fishbein GA, Preston SD. Revising the histopathologic definition of myocarditis: Birth of the seaport criteria. Cardiovascular Pathology. 2025;79:107765. 10.1016/j.carpath.2025.107765. 40819739

